# Pulmonary Rheumatoid Nodules: A Case Report

**DOI:** 10.1002/rcr2.70645

**Published:** 2026-06-05

**Authors:** Mingli Yuan, Wen Yin, Liangchao Wang, Shenglan Ye

**Affiliations:** ^1^ Department of Pulmonary and Critical Care Medicine Central Hospital of Wuhan, Tongji Medical College, Huazhong University of Science and Technology Wuhan China

**Keywords:** case report, pulmonary nodules, rheumatoid arthritis, rheumatoid nodules

## Abstract

Rheumatoid arthritis (RA) is an autoimmune disorder primarily targeting joints, with the lungs often being the most affected organ outside the joints. Pulmonary rheumatoid nodules (PRNs) are unusual manifestations of RA in the lungs. We report a case of a 74‐year‐old Chinese man with a smoking history, diagnosed with RA and treated with leflunomide and prednisone, who showed multiple peripheral or pleural nodules in both lungs on computed tomography (CT) scans. Pathological analysis of the right middle lobe lesion from CT‐guided percutaneous core needle biopsy showed lymphocytic and neutrophilic infiltration, eosinophilic necrosis, scattered multinucleated giant cells and no basophilic necrosis or vasculitis. The diagnosis of PRNs was made after thorough examinations and multidisciplinary consultation and tocilizumab was recommended for subsequent therapy. PRNs, a rare extra‐articular manifestation of RA, should be distinguished from infections and malignancies. High‐resolution chest CT and biopsy can help in the diagnosis.

## Graphical abstract

PRNs, a rare extra‐articular manifestation of RA, should be distinguished from infections and malignancies. High‐resolution chest CT and biopsy help in the diagnosis.

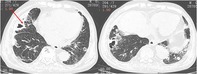

## 1 Introduction

Rheumatoid arthritis (RA) is an autoimmune disease primarily affecting joints, leading to inflammatory joint pain, swelling, stiffness and potential joint damage. It can also affect other organs, resulting in pulmonary, cardiac, cutaneous, neurological and ocular manifestations. Globally, RA affects about 0.5% of people, with the highest incidence in those aged 50–59 [1]. The lungs are the most commonly affected extra‐articular site, with involvement in about 60% of cases [2]. RA associated interstitial lung disease is the most prevalent pulmonary manifestation, followed by airway disease, pleural effusion and rheumatoid nodules. Pulmonary rheumatoid nodules (PRNs) are relatively uncommon clinical entities, detected in fewer than 1% of routine chest radiographs yet in approximately 30% of patients via computed tomography (CT) and lung biopsy [2, 3]. These lesions are frequently misdiagnosed as malignant tumours or infectious processes. We present a case of PRNs, highlighting its radiographic and pathological features to aid in accurate diagnosis.

## 2 Case Report

A 74‐year‐old Chinese man was hospitalised for a 3‐month cough and sputum production, along with 2 days of exertional shortness of breath. He has had RA for a year, treated with leflunomide and prednisone. He quit smoking 3 years ago after a 40 pack‐year history. Examination revealed a respiratory rate of 21, oxygen saturation of 95%, blood pressure of 118/76 and a heart rate of 90. There were no subcutaneous nodules or enlarged lymph nodes. Upon auscultation, bronchial breath sounds were detected in the right lobe, accompanied by coarse crackles bilaterally at the lung bases; the heart rhythm was regular and there was slight leg oedema.

**FIGURE 1:**
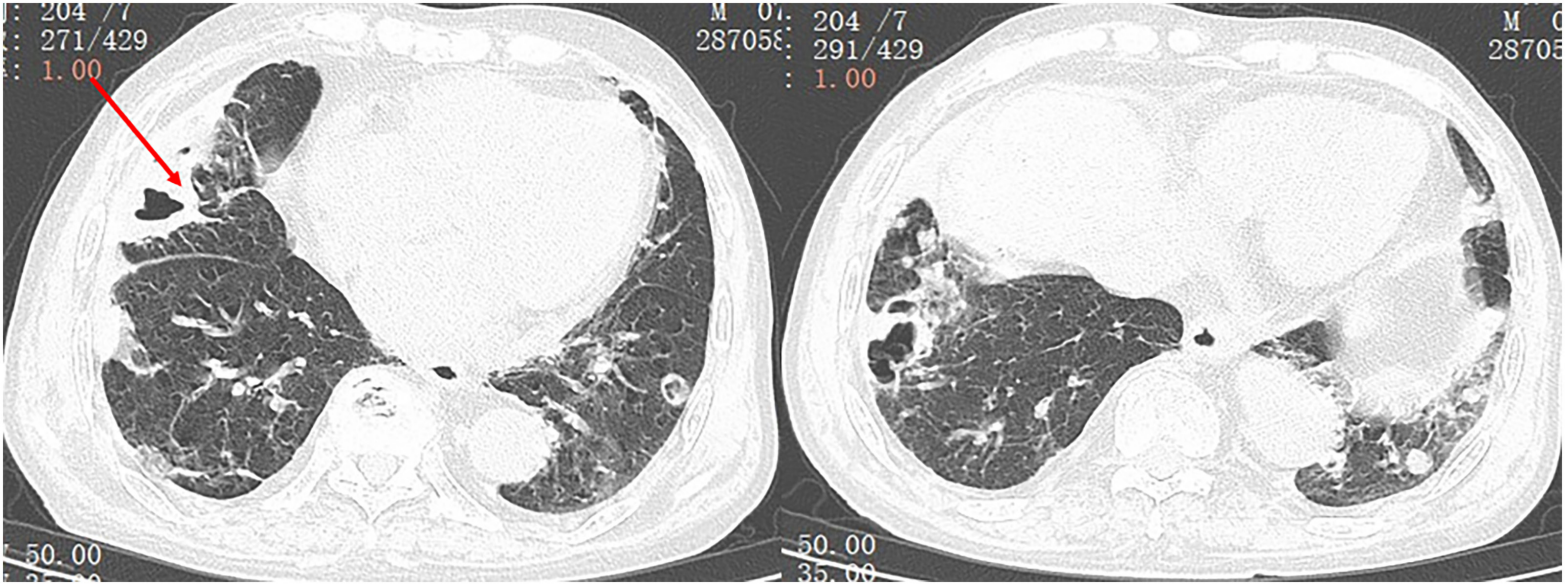
Thoracic computed tomography: Multiple peripheral or pleural based nodules in both lungs, ranging from a few millimetres to several centimetres, some with uneven‐walled cavities. A percutaneous needle biopsy was conducted on the arrow‐indicated lesion.

**FIGURE 2:**
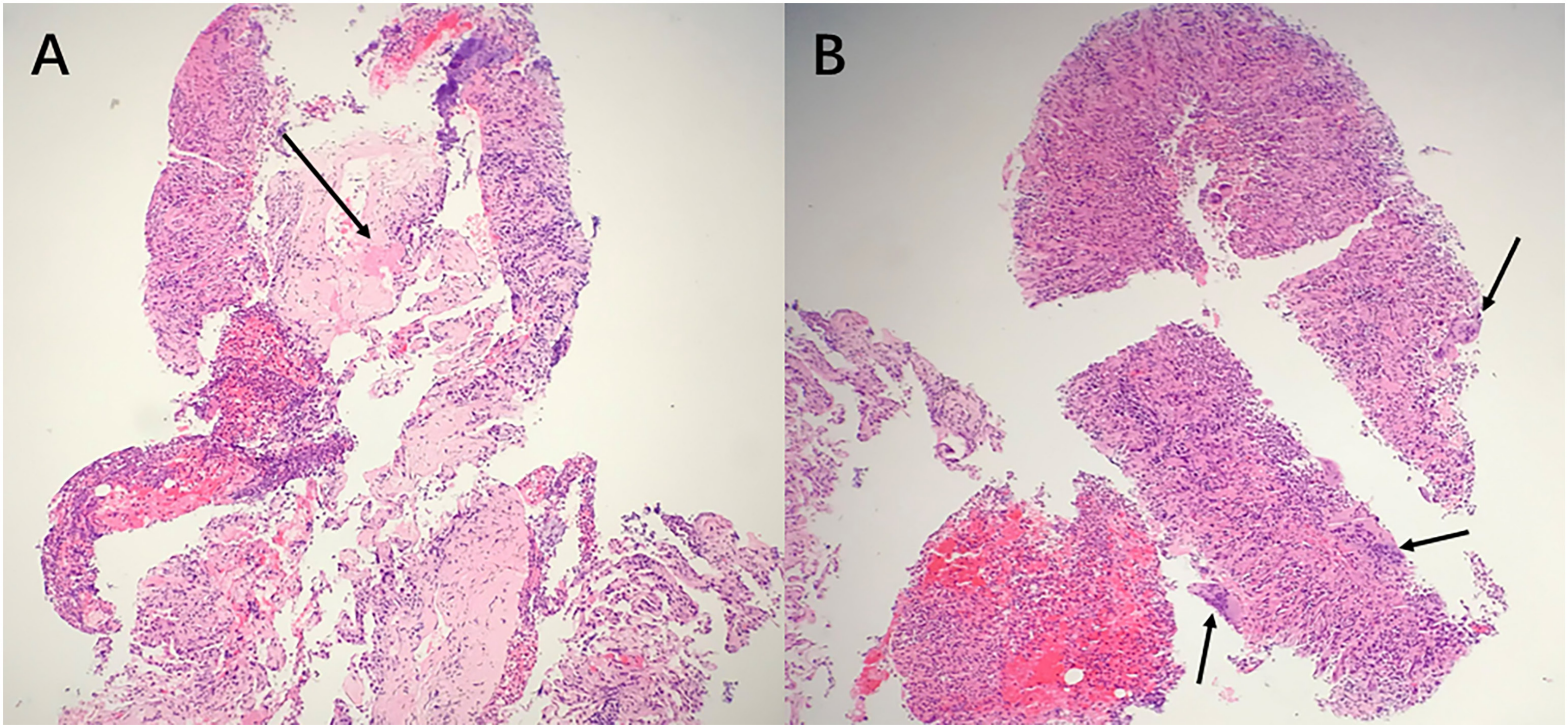
Pathology: Extensive neutrophil and lymphocyte infiltration (A and B), eosinophilic necrosis (arrow) (A) and scattered multinucleated giant cells (arrow) (B). (Haematoxylin and eosin stain [H&E], original magnification ×4).

Chest CT showed 18 bilateral pulmonary nodules, predominantly peripheral, mostly solid with partial subsolid features, measuring several millimetres to centimetres. Several of these nodules displayed irregular‐walled cavities and a cavitary nodule located in the right middle lobe was accompanied by surrounding consolidation (Figure 1). Key laboratory findings were as follows: haemoglobin 95 g/L, neutrophils 6.35 × 10^9^/L, high‐sensitivity C‐reactive protein (CRP) 58.3 mg/L, erythrocyte sedimentation rate (ESR) 45 mm/h, interleukin‐6 (IL‐6) 16.48 pg/mL, B‐type natriuretic peptide 921 pg/mL, rheumatoid factor 191 IU/mL, anti‐keratin antibody weakly positive, anti‐cyclic citrullinated peptide antibody > 400 IU/mL. Tests for lung tumour markers, anti‐neutrophil cytoplasmic antibodies, procalcitonin and sputum pathogens were negative. His 28‐joint Disease Activity Score (DAS28‐ESR) was 5.0, indicating moderate disease activity of RA. Pathological analysis of the right middle lobe lesion from CT‐guided percutaneous core needle biopsy showed lymphocytic and neutrophilic infiltration, eosinophilic necrosis and multinucleated giant cells, with no basophilic necrosis or vasculitis (Figure 2). Special stains (Periodic Acid‐Schiff, hexamine silver, acid‐fast) and targeted next‐generation sequencing were negative.

PRNs were diagnosed via multidisciplinary consultation. The patient had moderately active RA under corticosteroid‐leflunomide therapy, complicated by new‐onset PRNs and elevated IL‐6 and CRP. Tocilizumab was administered to rapidly suppress IL‐6‐mediated inflammation. The patient is now under follow‐up.

## 3 Discussion

Pulmonary involvement represents the most prevalent extra‐articular manifestation of RA, affecting up to 60% of patients. This condition may impact the lung parenchyma, manifesting as interstitial lung disease or rheumatoid nodules, the pleura, resulting in pleural effusion and the airways, leading to conditions such as cricoarytenoiditis or bronchiectasis [2]. The prevalence of PRNs ranges from under 1% in routine chest x‐rays to approximately 30% in CT scans and lung biopsies [2, 3].

Risk factors for the development of PRNs include longer disease duration, smoking history, elevated anti‐cyclic citrullinated peptide antibody and rheumatoid factor levels, male sex, genetic predisposition and severe joint disease [2, 3]. The exact mechanism of PRNs remains unclear, although they share similarities with the inflammatory processes in RA [4]. Paradoxically, PRNs can result from certain RA treatments [5] and may be accelerated by conventional synthetic as well as biologic disease‐modifying anti‐rheumatic drugs (DMARDs) like methotrexate, leflunomide, azathioprine and etanercept [4].

PRNs have pathological changes similar to subcutaneous rheumatoid nodules, including fibrinoid necrosis in the centre surrounded by palisading epithelioid cells along with infiltration of inflammatory cells [4]. In addition, infection and malignancy must be excluded as part of the diagnostic workup.

PRNs often appear as single or multiple nodules of varying sizes on radiographs, with about 50% developing cavities, usually in the superior and middle peripheral lobes or near the pleura [2, 5, 6]. They are usually asymptomatic unless complications like cavity formation, rupture or infection occur, leading to issues such as hemoptysis, pneumothorax, pleural effusion or bronchopleural fistula [2]. PRNs should be distinguished from infectious diseases such as pulmonary tuberculosis, septic embolism, pulmonary fungal infection, non‐infectious diseases such as sarcoidosis, vasculitis and neoplastic diseases. Diagnosing PRNs can be challenging, especially in RA patients on immunosuppressants, due to the prevalence of similar conditions and a 4.5% incidence of lung cancer in these patients [7]. Although Matthew et al. reported that optimal sensitivity (77%) and specificity (92%) for PRNs were achieved when ≥ 3 CT features (≥ 4 nodules, peripheral distribution, cavitation, satellite nodules, smooth margins and subpleural rind) were present [8], biopsy is generally performed more frequently for PRNs than for subcutaneous rheumatoid nodules [6].

The treatment of PRNs is not well established till now. PRNs do not reliably align with RA's clinical progression. They are unpredictable, potentially changing, regressing or staying the same despite treatment and may even worsen after starting RA therapies [5, 6]. Consequently, there is a recognised uncertainty concerning the optimal management strategies for patients with nodules exacerbated by RA medications and switching to drugs with less lung impact is advisable. The anti‐CD20 monoclonal antibody rituximab was reported to effectively decrease the number and the size of PRNs [9]. The Janus kinase (JAK) inhibitors tofacitinib and baricitinib were also proved to have a positive effect on PRNs [4, 5]. Tocilizumab is a monoclonal antibody directed against the IL‐6 receptor, was reported to prevent PRNs [10], while it is involved in the acceleration of subcutaneous rheumatoid nodule in a case series [11]. Surgical management could be considered for PRNs with a high risk of rupture and bleeding, as well as complications such as pneumothorax and bronchopleural fistula. The patient in our case exhibited elevated IL‐6 and C reactive protein (CRP) levels, indicating IL‐6 pathway activation, making him a good candidate for tocilizumab therapy. Studies confirm tocilizumab's efficacy for PRNs with no adverse effects and its subcutaneous form offers convenient long‐term use. Thus, tocilizumab was initiated and the patient is being closely monitored.

In conclusion, PRNs are an uncommon extra‐articular feature of RA, appearing as single or multiple nodules, sometimes with cavities and the pathological changes are similar to subcutaneous rheumatoid nodules. Diagnosing PRNs can be difficult and often requires a biopsy. It's important to conduct comprehensive tests to exclude infectious diseases, non‐infectious diseases and malignancies before the diagnosis of PRNs.

## Author Contributions

**Mingli Yuan:** conceptualization, formal analysis, writing – original draft, funding acquisition. **Wen Yin:** investigation, data curation. **Liangchao Wang:** investigation, data curation. **Shenglan Ye:** conceptualization, formal analysis, investigation, data curation, supervision, writing – review and editing.

## Funding

This work was supported by Wuhan Municipal Natural Science Foundation Exploratory Program, 2025020701020249.

## Consent

The authors declare that written informed consent was obtained for the publication of this manuscript and accompanying images using the consent form provided by the Journal.

## Conflicts of Interest

The authors declare no conflicts of interest.

## Data Availability

The data that support the findings of this study are available on request from the corresponding author. The data are not publicly available due to privacy or ethical restrictions.
